# A Novel *Acinetobacter baumannii* Bacteriophage Endolysin LysAB54 With High Antibacterial Activity Against Multiple Gram-Negative Microbes

**DOI:** 10.3389/fcimb.2021.637313

**Published:** 2021-03-02

**Authors:** Fazal Mehmood Khan, Vijay Singh Gondil, Changchang Li, Mengwei Jiang, Junhua Li, Junping Yu, Hongping Wei, Hang Yang

**Affiliations:** ^1^ CAS Key Laboratory of Special Pathogens and Biosafety, Center for Biosafety Mega-Science, Wuhan Institute of Virology, Chinese Academy of Sciences, Wuhan, China; ^2^ International College, University of Chinese Academy of Sciences, Beijing, China

**Keywords:** bacteriophage, endolysin, *Acinetobacter baumannii*, Gram-negative “superbugs”, antimicrobial resistance

## Abstract

The rapid spread and emergence of multidrug-resistant *Acinetobacter baumannii* and other pathogenic Gram-negative bacteria spurred scientists and clinicians to look for alternative therapeutic agents to conventional antibiotics. In the present study, an *A. baumannii* bacteriophage p54 was isolated and characterized. Morphological and genome analysis revealed that bacteriophage p54 belongs to Myoviridae family with a genome size of 165,813 bps. A novel endolysin, namely LysAB54, showing low similarity with other well-known related endolysins, was cloned, expressed, and characterized from the bacteriophage p54. LysAB54 showed significant bactericidal activity against multidrug-resistant *A. baumannii* and other Gram-negative bacteria, including *Pseudomonas aeruginosa*, *Klebsiella pneumoniae*, and *Escherichia coli*, in the absence of outer membrane permeabilizers. Based on all those observations, LysAB54 could represent a potential agent for the treatment of multidrug-resistant Gram-negative superbugs.

## Introduction

Bacterial infections deleteriously impact human health and are a leading cause of mortality worldwide. The emergence of antibiotic-resistant bacterial strains further exaggerates the present situation and alarming for humankind in various aspects of daily life. The occurrence of multi-drug resistant (MDR) Gram-negative bacterial pathogens, which mainly includes antibiotic-resistant strains of *Acinetobacter baumannii*, *Pseudomonas aeruginosa*, *Klebsiella pneumoniae*, and *Escherichia coli*, may lead to serious complications and nosocomial infections (Bonomo and Szabo, 2006; Spellberg et al., 2013; Heron, 2017; Collaborators, 2018). *A. baumannii* is an opportunistic Gram-negative pathogen that can initiate several serious nosocomial infections, which majorly includes wound infections, urinary tract infections, ventilator related pneumonia, and secondary meningitis. The high resistance of *A. baumannii* toward conventional antibiotics makes their management very cumbersome (Valencia et al., 2009; Pendleton et al., 2013; Potron et al., 2015). The high degree of antibiotic resistance conferred by *A. baumannii* and other pathogenic bacteria spurred scientists and clinicians to look for alternative therapeutic agents to antibiotics (Magiorakos et al., 2012).

Among various available alternative agents, bacteriophage encoded peptidoglycan hydrolases, popularly known as endolysins or lysins, represent a promising alternative for the treatment of drug-resistant bacteria. Endolysins are enzymatic proteins that are produced inside the bacterial host at the later stage of bacteriophage replication and can lyse bacterial hosts when applied externally (Fischetti, 2008; Nelson et al., 2012; Schmelcher et al., 2012; Yang et al., 2014; Gondil et al., 2020). Endolysin renders numerous advantages over other alternative antibacterial agents, including high activity against drug-resistant pathogens, low chances of resistance, and high specificity towards target bacterium (Loessner et al., 2002; Schuch et al., 2002; Fischetti, 2008; Briers et al., 2009). Several endolysin have been reported to harbor broad-spectrum activity against Gram-positive bacteria *in vitro* and *in vivo*, such as PlyC (Nelson et al., 2006), ClyR (Yang et al., 2015a), Cpl-1 (Loeffler et al., 2003), ClyJ (Yang et al., 2020), and ClyV (Huang et al., 2020).

On the Contrary, due to the presence of protective bacterial outer membrane, most endolysins fail to exhibit prominent activity against Gram-negative bacteria (Fischetti, 2005; Rodríguez-Rubio et al., 2016). However, treatment bacteria with outer membrane permeabilizers (OMPs), such as citric acid, trichloroethane (CHCl_3_), Triton X-100, and EDTA, can improve the antibacterial activity of endolysins and conquer the hindrance presented by the outer membrane of Gram-negative bacteria (Helander and Mattila-Sandholm, 2000; Briers et al., 2007; Briers et al., 2011; Briers et al., 2014; Yang et al., 2015b; Guo et al., 2017). In this context, several endolysins have showed improved or synergistic bactericidal activity against MDR *A. baumannii* and *P. aeruginosa* in the presence of OMPs, including LysSS (Kim et al., 2020b), Abtn-4 (Yuan et al., 2020), LysPA26 (Guo et al., 2017), PlyA (Yang et al., 2015b), ABgp46 (Oliveira et al., 2016), and OBPgp279 (Walmagh et al., 2012). However, lysins with OMP-independent antibacterial activity against Gram-negative microbes still un-adequately addressed since few of them are demonstrated effective in animal infection models.

In the present study, we report the comprehensive characterization of a novel *A. baumannii* bacteriophage p54 and its endolysin, namely, LysAB54. Our results demonstrated that LysAB54 has a high and rapid antibacterial activity against a range of antibiotic-resistant Gram-negative bacterial strains in the absence of OMPs.

## Materials and Methods

### Collection of Water Sample for Bacteriophage Isolation

The water sample was collected in sterile 50 ml screw tubes from the Tongji Hospital, Wuhan, Hubei Province, China. The water samples were kept in an ice bucket box to preserve the specimen. After removing the impurities by sterile syringe filter, samples were kept in 4°C for further use.

### Isolation of Bacteriophage p54

The stored water sample was mixed 1:1 with phage buffer (50 mM Tris-HCl, 150 mM NaCl, 10 mM MgCl_2_, 2 mM CaCl_2_, pH 7.5). The *A. baumannii* strain WHG3083 was cultured in lysogeny broth (LB) at 37°C to logarithmic phase and then transferred to the conical flask. Conical flask solution containing water sample, phage buffer, and bacteria were incubated aerobically in shaking incubator on 160 rpm at 37°C for 2–3 days. After incubation, solution was centrifuged (1,000 rpm for 10 min, 4°C) and filtered through a sterile 0.22 µm syringe filter (Guangzhou Jet bio-filtration Co., Ltd). Then, 500 μl of the filtrate was mixed with 500 μl of *A. baumannii* in a sterile 1.5 ml microcentrifuge tube and incubated aerobically in a shaking incubator on 160 rpm at 37°C for 15–20 min. The mixture was mixed with 4 ml of 0.7% soft agar (approximately 50°C) in a sterile 15 ml tube (Guangzhou Jet bio-filtration Co., Ltd) and poured onto the LB agar plates and incubated overnight at 37°C.

### Purification of the Bacteriophage p54

A single plaque was picked and resuspended in the phage buffer in a sterile 1.5 microcentrifuge tube. The microcentrifuge tube containing plaque was vortexed for approximately 2–3 min. The vortexed solution was further filtered through a sterile 0.22 µm syringe filter. 10 μl of the filtered solution (10-fold serially diluted) was mixed with the 1 ml of *A. baumannii* cell suspension and incubated on 160 rpm at 37°C for 15–30 min. Afterwards, bacterium-phage mixture was mixed with 4 ml of soft agar and poured onto LB agar plates and incubated overnight at 37°C. This cycle was repeated for five times to obtain purified bacteriophage p54.

### Bacteriophage p54 Genome Extraction and Sequencing Analysis

The bacteriophage p54 genome was extracted and sequenced following the protocol and procedures as previously described (Kering et al., 2020). The sequenced genome was compared with the available whole genome sequences of *A. baumanni*i bacteriophages by the Neighbor-Joining method (Saitou and Nei, 1987). The bootstrap consensus tree inferred from 1,000 replicates is taken to represent the evolutionary history of the taxa analyzed (Felsenstein, 1985). Branches corresponding to partitions reproduced in less than 50% bootstrap replicates are collapsed. The percentage of replicate trees in which the associated taxa clustered together in the bootstrap test (1,000 replicates) are shown next to the branches (Felsenstein, 1985). The evolutionary distances were computed using the p-distance method and are in the units of the number of base differences per site. This analysis involved 10 nucleotide sequences and the codon positions included were 1st+2nd+3rd+Noncoding. All ambiguous positions were removed for each sequence pair. There was a total of 16,5813 positions in the final dataset. Evolutionary analyses were conducted in MEGA X software (Kumar et al., 2018).

### Transmission Electron Microscopy (TEM) of the Bacteriophage p54

The bacteriophage p54 was purified and concentrated using cesium chloride density gradient ultracentrifugation on 35,000 rpm for 2 h at 4°C. The concentrated bacteriophage was stained with freshly prepared phosphotungstic acid solution (2%) for 3 min and spot dried on the copper grid for 2 h. The samples were examined on H-7000FA transmission electron microscope (Hitachi, Japan).

### Bacterial Culture Conditions

All bacterial strains were cultured in LB broth aerobically at 37°C. The stocks of bacterial strains containing 20% glycerol was store in -80°C refrigerator for long time storage. *E. coli* BL21(DE3) (Takara) cells were used for cloning and protein expression.

### Cloning of LysAB54 in the Expression Vector

The open-reading frame (ORF) encoding the putative endolysin LysAB54 was amplified by Polymerase Chain Reaction (PCR) with the primers (5′ to 3′): 54-Forward: TATACCATGGACGTTAAACCATTTTTTG (*Nco*I) and 54-Reverse: TATACTCGAGTTGTTCAAAATACGCTTTCTC (*Xho*I). The purified (Omega Bio-tek^®^) PCR product was digested with restriction endonuclease enzymes *Nco*I and *Xho*I (Thermo Scientific). Purified PCR product was ligated into pET28a (+) and transformed to the competent *E. coli* BL21(DE3) cells. The inserted gene was further confirmed by Sanger sequencing (Sangon Biotech, Shanghai).

### Protein Expression and Purification

*E. coli* BL21 (DE3) containing the pET28a-LysAB54 was grown in 500 ml LB with 100 mg/ml of kanamycin for approximately 2–3 h until the OD_600_ reached to 0.5–0.6. Protein expression was induced with 1 mM Isopropyl-beta-D-thiogalactopyranoside (IPTG; Thermo Scientific) at 16°C for 16 h. The bacterial culture was harvested by centrifugation at 10,000 rpm at 4°C for 10 min. Bacterial cells were washed once by PBS (pH7.4) and centrifuged at 10,000 rpm at 4°C for 10 min. Cells were disrupted through sonication in ice cold environment. The insoluble cell fragments were removed through centrifugation at 10,000 rpm at 4°C for 10 min and the supernatant was collected by filter through 0.22 µm syringe filter. His-tagged LysAB54 was purified using nickel nitrilotriacetic acid affinity column chromatography (GE Healthcare, US) with gradient of imidazole solutions. After purification, proteins were dialyzed overnight at 4°C against Tris buffer (pH 7.4). The purity of protein was assessed using SDS-PAGE.

### Antibacterial Activity Assay of Endolysin LysAB54

Antibacterial activity of endolysin LysAB54 was determined as defined previously (Yang et al., 2015b) with minor modifications. To determine the antibacterial activity of LysAB54, the bacteria were cultured in LB broth at 37°C for both the logarithmic phase (OD_600_ = 0.6–0.8) and stationary phase (OD_600_ = 1.4–1.6). Bacterial culture was centrifuged at 8,000 rpm at 4°C for 5 min and bacterial cells were washed and re-suspended in Tris buffer (pH 7.4). LysAB54 was added into bacterial suspension and incubated aerobically at 37°C for 60 min. Groups treated with the same amount of Tris buffer instead of LysAB54 were used as negative controls. The mixture was then serially diluted than plated on the LB agar petri dishes, incubated overnight aerobically at 37°C.

### Killing Range of LysAB54

To evaluate the bactericidal activity of endolysin LysAB54 against clinical Gram-negative isolates, logarithmic phase bacteria were treated with 100 μg/ml of LysAB54 at 37°C for 1 h. The reduction in bacterial count in term of LogCFU/mL was defined as the antibacterial activity of LysAB54 against tested isolate.

### The Activity of LysAB54 in Complex Medium

To test the antibacterial activity of endolysin LysAB54 against *A*. *baumannii* in complex medium, logarithmic phase bacteria were re-suspended in LB broth and human-serum (Sigma Aldrich, Shanghai, China), and treated with 100 μg/ml of LysAB54 at 37°C for 1 h. The mixture was serially diluted and plated on LB agar Petri dishes for viable cell count.

### Statistical Analysis

The data were analyzed using GraphPad prism Version 5 software on Microsoft Windows 2019 operating system. All the experiments were performed in triplicates on different time points. The data was expressed in term of Mean ± Standard Deviation (SD) for statistical considerations.

## Results

### Characterization of Bacteriophage p54

**Figure 1:**
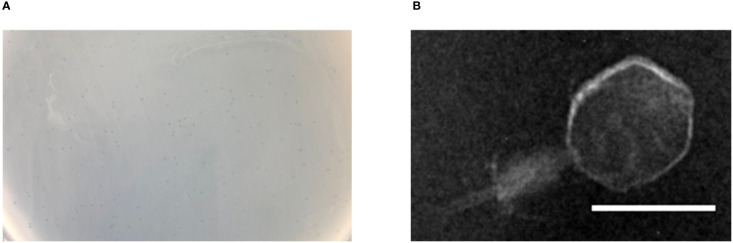
Characteristics of bacteriophage p54. **(A)** Double layer agar assay of the isolated bacteriophage p54 with host bacterium. **(B)** Morphological analysis of the bacteriophage p54 by transmission electron microscopy (TEM). Scale bar: 25 µm.

Bacteriophage p54 was isolated and purified from the hospital water sample. Double-layer agar assay showed clear plaques on *A. baumannii* lawn postulating the lytic potential of isolated bacteriophage p54 (**Figure 1A**). Morphological analysis showed that the bacteriophage p54 contains an icosahedral head and a short tail, with a particle size of 24.4×55.8 nm (**Figure 1B**). Based on these observations, bacteriophage p54 can be classified to the family of Myoviridae according to the latest classification by the International Committee on the Taxonomy of Viruses (ICTV).

### Genome Sequencing and ORF Annotation of Bacteriophage p54

**Figure 2:**
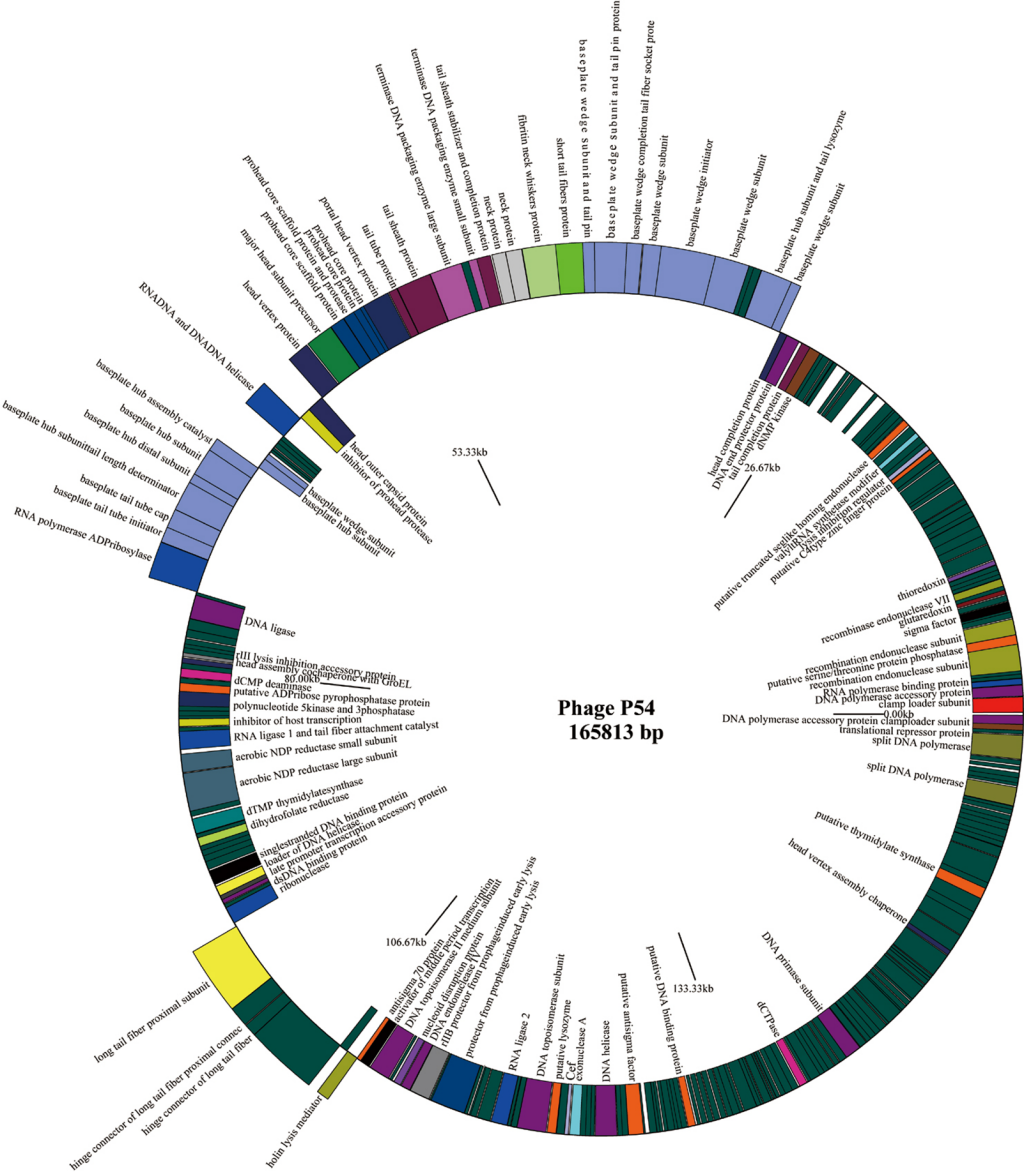
Genome map of the bacteriophage p54. Genes on the positive chain of the genome (Outer ring); Gene on the negative chain of the genome (Inner ring). The genome direction from 5′ to 3′ in the figure is drawn counterclockwise. Functions of putative open-reading frames (ORFs) are marked.

Full genome sequencing analysis showed that bacteriophage p54 consists 165,813 bps with a GC content of 36.3%. A Blast-P analysis further revealed that bacteriophage p54 contains 248 predicted ORFs, with putative functions categorized into multiple functional modules, including phage structure, host lysis, phage DNA metabolism and modifications (**Figure 2**). Whole-genome based phylogenetic analysis showed that bacteriophage p54 possesses maximum similarity with *A. baumannii* phage AP22 (NCBI No: HE806280) among various phages analyzed (**Figure S1**), indicating the novelty of bacteriophage p54.

### Bioinformatics Analysis of the Endolysin LysAB54

**Figure 3:**
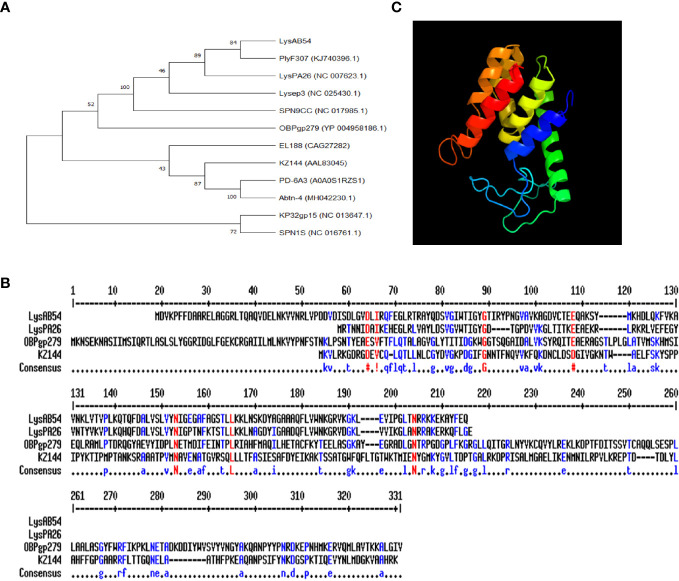
Phylogenetic analysis of LysAB54. **(A)** Phylogenetic relationship of various endolysins. The neighbor-joining trees were based on the ClustalW alignment of DNA sequences by MEGAX software. **(B)** Multi-alignment analysis of LysAB54 with other closely related Gram-negative endolysins. **(C)** Predicted three-dimensional structure of LysAB54. Three‐dimensional structure model by Phyre2 web server. NORMAL mode. Confidence in the model: 100.0%. 148 residues (79% of sequence) have been modeled with 100.0% confidence by the single highest scoring template. Model dimensions (Å): X: 35.277, Y: 41.465, Z: 46.142.

The putative endolysin LysAB54 of bacteriophage p54, i.e., ORF159 (120,967–121,530 bp), comprised 187 amino acids with a putative lysozyme activity. As shown in **Figure 3A**, a phylogenetic analysis of LysAB54 and other Gram-negative endolysins showed that LysAB54 has similarity with the other reported endolysin containing lysozyme domain, such as LysPA26 of *A. baumannii* phage (Guo et al., 2017). The similarity of LysAB54 with other closely related endolysins was further performed by the MultAlign server and revealed a lower level of similarity with known related endolysins, postulating the novelty of LysAB54 (**Figure 3B**). Phyre2 server-based structural prediction showed that LysAB54 contains multiple alpha-helix domains with the absence of predictable beta plated sheets (**Figure 3C**).

### LysAB54 Shows High Bactericidal Activity Against *A. baumannii*

**Figure 4:**
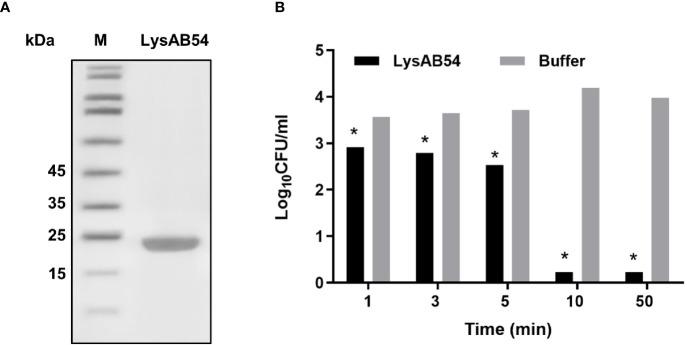
Characteristics of LysAB54. **(A)** Purified LysAB54 on SDS-PAGE gel. Lane 1 showing LysAB54 and lane 2 showing protein marker. **(B)** Time-killing activity of LysAB54 against logarithmic phase of *A. baumannii* WHG 40090 in Tris buffer. Data are shown as means ± standard deviations. *p < 0.05.

SDS-PAGE analysis showed a single band of LysAB54 after purification (**Figure 4A**), which was consistent with its predicted molecular mass of 24 kDa. The time-killing assay showed that the LysAB54 (100 μg/ml) could kill logarithmic *A. baumannii* with 0.6 logs of reduction in the first 1 min of incubation, while up to 4 logs of decrement in bacterial count was achieved after incubation for 10 min. These results showed that the LysAB54 exhibits robust as well as rapid bactericidal activity (**Figure 4B**).

### Antibacterial Activity of LysAB54 Against Logarithmic Phase Gram-Negative Bacteria

**Figure 5:**
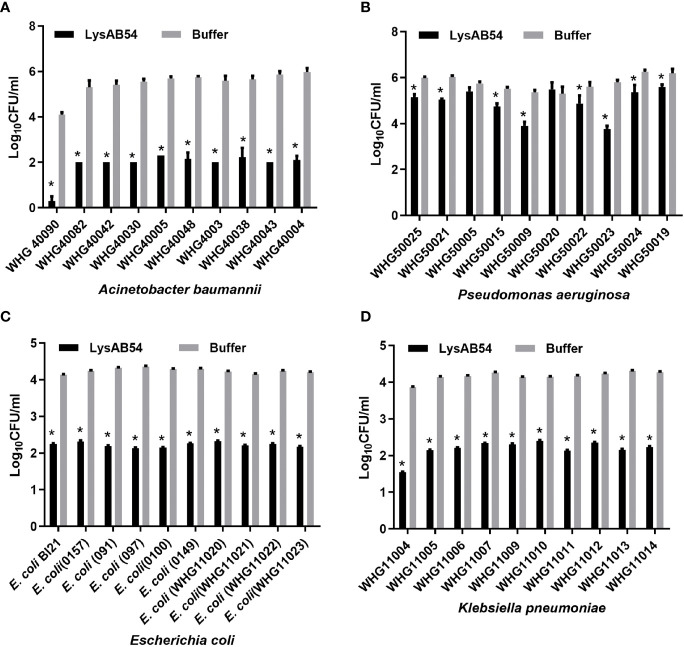
Antibacterial activities of LysAB54 against logarithmic phase of Gram-negative microbes in Tris buffer. Logarithmic phase strains of *A baumannii* **(A)**, *P. aeruginosa* **(B)**, *E. coli* **(C)**, and *K pneumoniae* **(D)** were treated with 100 μg/ml of LysAB54 for 1 h at 37°C. The viable cell number was calculated on the LB agar plates. Data are shown as means ± standard deviations. *p < 0.05.

The antibacterial activity of LysAB54 in Tris buffer against each ten different clinical isolates of *A. baumannii*, *P. aeruginosa*, *E. coli*, and *K. pneumoniae* was investigated. As shown in **Figure 5**, LysAB54 showed high bactericidal activity against all isolates of *A. baumannii*, *E. coli*, and *K. pneumoniae* tested. Moreover, 8 out of 10 P*. aeruginosa* strains were found to be highly susceptible to LysAB54 (**Figure 5B**). The difference in antibacterial activity of log reduction against these clinical strains may be due to the difference in the molecular architecture of the bacterial outer membrane. Notably, reduction of 4 logs (from 4.2 to 0), 2.17 logs (from 5.77 to 3.60), 2 logs (from 4.22 to 2.16), and 2.33 log (from 3.86 to 1.53) were observed in *A. baumannii* WHG 40090, *P. aeruginosa* WHG50023, *E. coli* WHG11023, and *K. pneumoniae* WHG11004, respectively. These results suggested that LysAB54 has a wide range of antibacterial activity against multi-drug resistant Gram-negative microbes.

### Antibacterial Activity of LysAB54 Under Various Conditions

**Figure 6:**
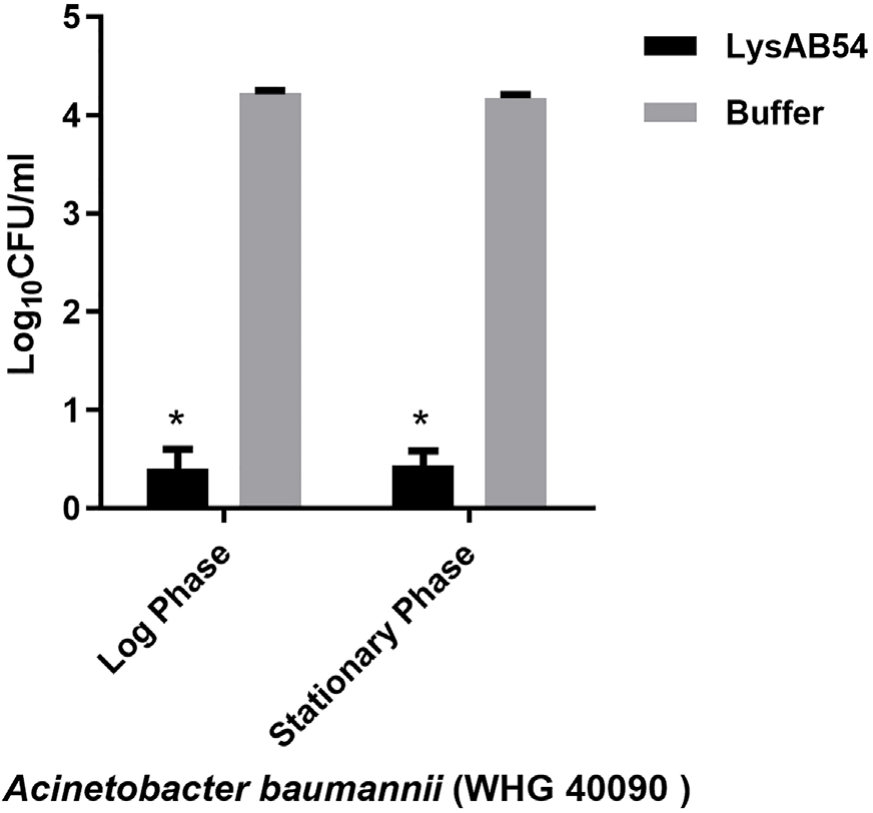
Antibacterial activities of LysAB54 against the logarithmic and stationary phase of *A. baumannii*. *A. baumannii* WHG 40090 cells under different growth phases were treated with 100 μg/ml of LysAB54 for 1 h at 37°C. The viable cell number was calculated on the LB agar plates. Data are shown as means ± standard deviations. *p < 0.05.

Previous reports showed that multiple Gram-negative endolysins have good activity against the logarithmic phase bacteria but less or rare active against the stationary bacteria (Yang et al., 2015b; Guo et al., 2017). Therefore, we first evaluated the bactericidal activity of LysAB54 with bacteria under different growth phases. Results showed that LysAB54 showed similar antibacterial activity against both logarithmic as well as stationary phase of *A. baumannii* (**Figure 6**). These results indicates that the bacterial phase have minor effect on the antibacterial activity of LysAB54.

Another challenge for application of Gram-negative endolysin is the reduced or abolished killing activity in a complex medium like serum. Most of the reported endolysins lose their activity in serum. In this context, we checked out the antibacterial activity of LysAB54 against the logarithmic phase of *A. baumannii* in complex medium, including LB broth and human serum. As reported previously, insignificant bactericidal activity of LysAB54 was observed in the complex mediums (**Figure S2**). The possible reason for loss of bactericidal activity is the ion exchange in these complex media which can potentially neutralize the interaction between endolysin and the negatively charged outer membrane.

## Discussion

The increasing infections caused by drug-resistant Gram-negative bacteria is a public health concern worldwide (Chopra et al., 2008; Scallan et al., 2011; Gondil et al., 2019). The Gram-negative bacterium *A. baumannii* which is responsible for multiple infections is an alarming threat to human health. Due to its intrinsic resistance and the overuse of antibiotics, some isolates of *A. baumannii* are now resistant to almost all known antibiotics (Manchanda et al., 2010). The increasing trend of antibiotic resistance in pathogenic bacteria necessitate the search for alternative antimicrobial agents to treat and cure such resistant pathogens mediated infections (Magiorakos et al., 2012). Currently, a very limited solutions are available in the treatment of MDR *A. baumannii* in clinical settings (Fischetti, 2010). Endolysin is a class of novel antimicrobial agents to treat and cure antibiotic-resistant bacteria due to its rapid bactericidal activity and low chances of antibiotic resistance (Nelson et al., 2012). Currently, several endolysins are being evaluated in clinical trials to explore their potential in treating Gram-positive bacterial infections (Kim et al., 2018). On the contrary, endolysins for Gram-negative bacterial infections are still under development. Although few studies have reported positive results in treating infections caused by Gram-negative bacteria *in vitro* and *in vivo*, including *A. baumannii* and *P. aeruginosa* (Ghose and Euler, 2020).

In this study, we characterized a novel bacteriophage endolysin LysAB54 encoded by a novel *A. baumannii* bacteriophage p54, which showed a broad range of antibacterial activity against Gram-negative bacteria irrespective of their growth phases, suggesting that the naturally occurring endolysin could be a promising alternative antimicrobial agent against number of MDR Gram-positive pathogens.

One excellent merit of the LysAB54 is its robust bactericidal activity against both logarithmic and stationary phase of Gram-negative bacteria in the absence of OMPs. Encouragingly, a reduction of over 4 logs in viable bacterial number was observed after treatment of *A. baumannii* cells with 100 μg/ml LysAB54 for 10 min.

To our knowledge, it is unconventional for an endolysin to render such rapid antibacterial activity against various antibiotic-resistant Gram-negative bacteria. Several natural known endolysins also exhibited antibacterial activity against Gram-negative bacteria in the absence of OMPs, such as OBPgp279 against *P. aeruginosa*, PlyF307 and LysAB21 against *A. baumannii* (Lai et al., 2011; Walmagh et al., 2012; Lood et al., 2015). It is demonstrated with increasing evidence that several endolysins are active against multiple Gram-negative bacteria, and few of them are reported active against Gram-positive pathogens *in vitro*, such as Abtn-4 (Yuan et al., 2020), LysSAP26 (Kim et al., 2020a), and LysSS (Kim et al., 2020b). Such lysins with broad host-range are commonly contain a lysozyme or glycoside hydrolase catalytic activity, which cleaves the β-1,4-glycosidic linkage between N-acetylmuramic acid (NAM) and N-acetylglucosamine (NAG) in peptidoglycans that is generally shared by various bacteria (Ghose and Euler, 2020). In this context, LysAB54 was predicted to contain a lysozyme activity, which is possibly the reason for its broad antibacterial activity against several other antibiotic-resistant Gram-negative bacteria, which includes *P. aeruginosa*, *K. pneumoniae*, and *E. coli*.

It is well known that environmental factors such as salts and proteins have a significant effect on the activity of Gram-negative endolysins. Our results showed that LysAB54 completely loses its antibacterial activity in the complex medium like LB broth and serum, which narrows down its application to topical infections, such as burn wound, or suture infections. One possible reason for the loss of LysAB54 activity in these media may be due to ions exchanging of endolysin in these environments, which could possibly neutralize the antibacterial activity of endolysins (Yang et al., 2015b).

In conclusion, our study demonstrates the morphological and genomic characteristics of a newly isolated *A. baumannii* bacteriophage p54 and the bactericidal activity of its endolysin, LysAB54, against multiple drug-resistant Gram-negative bacteria under various growth phases and conditions. The OMP-independent activity of LysAB54 makes it a potential candidate for the treatment of infections caused by multiple drug resistant Gram-negative pathogens. However, molecular dissection and validation of LysAB54 in biofilms and animal models still need to be established to reveal the enigmatic aspects of the present endolysin.

## Data Availability Statement

The original contributions presented in the study are included in the article/**Supplementary Material**. Further inquiries can be directed to the corresponding authors.

## Author Contributions

HY and HW designed the study. FK, VG, CL, MJ, and JL performed the experiments. FK, VG, JY, and HY performed data analysis. HY and HW contributed with reagents and/or funds for research. FK and VG wrote the draft manuscript. HY revised the manuscript. All authors contributed to the article and approved the submitted version.

## Funding

This work was financially supported by the Youth Innovation Promotion Association CAS (to HY) and the National Natural Science Foundation of China (no. 31770192, no. 32070187 to HY).

## Conflict of Interest

The authors declare that the research was conducted in the absence of any commercial or financial relationships that could be construed as a potential conflict of interest.
