# Supplementary material for: A Novel Acinetobacter baumannii Bacteriophage Endolysin LysAB54 With High Antibacterial Activity Against Multiple Gram-Negative Microbes
Source: Front Cell Infect Microbiol. 2021 Mar 2;11:637313. doi: 10.3389/fcimb.2021.637313 (PMC7960757; doi:10.3389/fcimb.2021.637313)
Supplement: Supplementary file 1 [file DataSheet_1.docx]

**Supplementary materials**

**A novel *Acinetobacter baumannii* bacteriophage endolysin LysAB54 with high antibacterial activity against multiple Gram-negative microbes**

Fazal Mehmood Khan^1,2^, Vijay Singh Gondil^1^, Changchang Li^1,2^, Mengwei Jiang^1^, Junhua Li^1^, Junping Yu^1,2*^, Hongping Wei^1,2*^, Hang Yang^1,2*^

**Table S1. Bacterial strains used in this work.**

|  | ^a^*A.baumannii* | ^b^*P.aeruginosa* | ^c^*K.pneumoniae* | ^d^*E. coli* |
| --- | --- | --- | --- | --- |
| 1 | WHG 40090 | WHG50025 | WHG11004 | E. coli BL21 |
| 2 | WHG40082 | WHG50021 | WHG11005 | E. coli (0157) |
| 3 | WHG40042 | WHG50005 | WHG11006 | E. coli (091) |
| 4 | WHG40030 | WHG50015 | WHG11007 | E. coli (097) |
| 5 | WHG40005 | WHG50009 | WHG11009 | E. coli (0100) |
| 6 | WHG40048 | WHG50020 | WHG11010 | E. coli (0149) |
| 7 | WHG4003 | WHG50022 | WHG11011 | E. coli (WHG11020) |
| 8 | WHG40038 | WHG50023 | WHG11012 | E. coli (WHG11021) |
| 9 | WHG40043 | WHG50024 | WHG11013 | E. coli (WHG11022) |
| 10 | WHG40004 | WHG50019 | WHG11014 | E. coli (WHG11023) |

^a^ obtained from Tongji Hospital, Wuhan, China.

^b^ obtained from Zhongnan Hospital of Wuhan University, Wuhan, China.

^c^ obtained from Shenzhen People's Hospital, Wuhan, China.

^d^ isolated from Animal Center of Wuhan Institute of Virology, Chinese Academy of Sciences, Wuhan, China.

**Figure S1.** Phylogenetic relationships of whole genome sequences of 10 bacteriophages. The neighbor-joining trees were based on the ClustalW alignment of DNA sequences by MEGAX software.


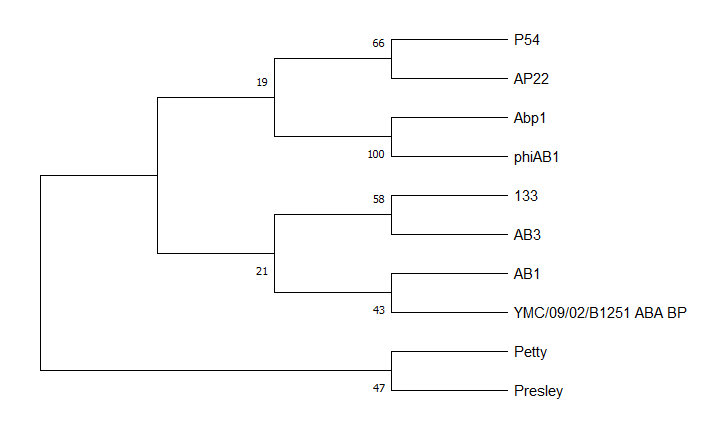


**Figure S2.** Antibacterial activity of LysAB54 in Complex medium LB broth and serum. Logarithmic *A. baumannii* cells resuspended in LB and human serum were treated with 100 μg/ml of LysAB54 for 1 h at 37°C. The viable cell number was calculated on the LB agar.
